# Patients with complex proximal tibial fractures overestimate the prognosis of their injury

**DOI:** 10.1007/s00068-021-01644-w

**Published:** 2021-03-15

**Authors:** Lena Keppler, Alexander Martin Keppler, Christoph Ihle, Philipp Minzlaff, Julian Fürmetz, Markus Beck, Tim Saier

**Affiliations:** 1 Department of Trauma Surgery, BG Trauma Center Murnau, Murnau, Germany; 2 Department of General, Trauma and Reconstructive Surgery, LMU Munich University Hospital, Munich, Germany; 3 Department of Traumatology and Reconstructive Surgery, BG Trauma Center Tuebingen, Tuebingen, Germany; 4 Department of Trauma and Orthopedic Surgery, Hospital Agatharied, Hausham, Germany; 5 Department of Orthopedic Surgery, Klinikum Rechts der Isar, Munich, Germany

**Keywords:** Tibial fracture, Osteosynthesis, Expectation, Knee function, Return-to-work, Return-to-sports

## Abstract

**Purpose:**

To investigate, if patients with complex proximal tibial fracture have realistic expectations on open reduction and internal fixation.

**Methods:**

114 patients (mean 49 years, SD ± 13) with closed AO-type B and C proximal tibial fracture were grouped (group B, respectively C). Prior to surgery expectations concerning knee function, pain, return to work/sports, and the risk for osteoarthritis was assessed with the Hospital for Special Surgery-Knee Surgery Expectations Survey (HFSS-KSE) and a non-validated ten-item survey.

**Results:**

92% of patients expected at least an almost natural knee postoperatively. All items regarding restoring knee function were ranked to be at least important in both groups. 65% in group B and 47% in group C expected at most occasional pain. 83% in group B and 67% in group C expected full return to work without any limitations. Patients with low physical work intensity expected significantly shorter incapacity to work in both groups (7.8, respectively 8.9 weeks). 71% in group B and 60% in group C expected to return to sports with at most small limitations. 33% in group B and 22% in group C assumed risk for osteoarthritis will be prevented by surgery.

**Conclusion:**

Expectations on surgery for complex proximal tibial fracture are high regardless of fracture type. The prognosis of many health and lifestyle domains was overestimated. The risk for osteoarthritis was underestimated. This study should sensitize surgeons to discuss realistic expectations. This may help to improve patient comprehension what leads to sensible expectations, resulting in improved patients´ satisfaction.

**Level of evidence:**

IV.

**Trial registration number:**

14104, Date of registration: 06/2015.

## Introduction

Proximal tibial fractures are reported with about 1% of all fractures [1]. Open reduction and internal fixation (ORIF) is considered the gold standard for partial (AO-type 41-B) or complete articular fractures (AO-type 41-C) [2]. To allow full range of motion, restoration of limb alignment and articular surface are the main goals of the surgical strategy.

Treatment of such injuries is challenging. Previous studies showed that proximal tibial fracture frequently results in residual pain, functional limitations, and osteoarthritis (OA) [3, 4]. Patients may not fully comprehend the severity of the fracture and the predicted outcome. Previously, several studies in orthopaedic surgery showed that patients have unrealistic expectations of surgical outcome [5, 6]. But, it remains unclear what patients expect from surgery for proximal tibial fracture. This information is important, since if expectations are not fulfilled, even in a trauma patient, dissatisfaction of the patient may result [7]. To provide a framework for setting reasonable goals resulting in improved patient-reported satisfaction, knowledge of patients´ comprehension concerning postoperative outcome is essential to direct patient education and shared decision-making.

The aim of this study was to preoperatively assess patients´ expectations of surgical outcome for complex proximal tibial fracture. It was hypothesized, that expectations are high regarding restoring knee function, pain relief, return-to-work and/or sports regardless of fracture type, demographic data and physical workload. Secondary, it was hypothesized, that patients underestimate their risk for secondary OA.

## Materials and methods

This prospective study was conducted at three Level-1 trauma centres. 114 consecutive patients with proximal tibial fracture were enrolled (01/2018–04/2019), with 58 male and 56 female patients. The mean age was 49 years (SD ± 13). Inclusion criteria were: closed AO-type B or C proximal tibial fracture after high energy trauma, indication for ORIF, minimum age 18 years, maximum age 70 years. Exclusion criteria were: open fractures, psychological comorbidities, chronic pain, alcoholism, diabetes, rheumatoid arthritis, and ligament reconstruction. The treatment of tibial plateau fractures was performed according to the AO principles. Surgical consent of patients was performed in all three participating hospitals by 3 surgeons who were at the end of their residency training, or were already board-certified orthopedic surgeons. A standardized information sheet was used in all study centers for surgical information. Surveys (see below) were handed out after written surgical consent was obtained.

In addition, epidemiological data, body mass index (BMI), employment, and sporting activity were recorded. Written informed consent was obtained from all patients prior to evaluation. Institutional review board approval for this study was obtained (blinded for review).

### Survey of patients’ expectation

Fractures were classified by the AO-classification using radiographs in two planes and CT scans.

Expectations were assessed with the Hospital for Special Surgery-Knee Surgery Expectations Survey (HFSS-KSE). This validated survey consists of twenty-items regarding areas of symptom relief and improvement of physical and psychosocial function. These items are rated on a four-point Likert scale, range from 1 (very important) to 4 (not important) [8].

To assess patient comprehension of surgical treatment, a non-validated ten item survey was used [9]. The items relate to the duration of occupational and sports disability, residual pain, comparison of the injured knee to a healthy knee, risk of secondary OA, and need for conversion to total knee arthroplasty (TKA). A healthy natural knee was defined as a joint with a normal functional status, i.e. free range of motion without pain at work or rest, and no limitations in activities of daily life due to pain. To record physical workload, REFA classification (Association for work design/work structure, industrial organization, and corporate development, formerly known as Reichsausschuss Für Arbeitszeitermittlung) was used [9]. The REFA classification describes the physical workload and is divided into four degrees (0 = no physical workload, 4 = very high physical workload).

### Statistical analysis

For statistical analysis IBM SPSS (Version 24, IBM, USA) was used. To analyse demographic data Mean, Median and Standard Deviation (SD) were measured. Normal distribution of data was tested and parametric test (*t*-test) was used. To show correlation, Spearman-Rho (*r*) was used. The level of significance was set at *p* ≤ 0.05. A power analysis was not performed due to a lack of comparative data.

## Results

### Patients’ characteristics

51 patients (45%) suffered an AO-type B fracture (group B) and 63 patients (55%) a type C fracture (group C). In total 16 patients were retired. Four of these patients were under the age of 65.

For details of group characteristics see Table 1.

**Table 1:** Patients’ characteristics and demographics

Characteristic	AO-B fracture (Group B)	AO-C fracture (Group C)	*p* value
Total (*n*)	51	63	
Male/female (*n*)	26/25	32/31	0.98
Mean age (years)	48 ± 14	50 ± 13	0.52
Mean body weight (kg)	75.4	76.8	
BMI (kg/m^2^)	25 ± 5	25 ± 4	0.69
Employed (*n*)	42	56	0.32
Retired (*n*)	9	7	0.32

Patients’ characteristics were similar in both groups

### Restoring knee function after surgical intervention

All patients had high expectations concerning surgical outcome (HFSS-KSE), regardless of fracture type. For most patients, every item was at least “important”. For both groups the items with the highest priorities were “pain relief” (1.19, respectively 1.18) and “improve ability to walk” (each 1.19). For details see Tables 2 and 3.

**Table 2:** Hospital for Special Surgery-Knee Surgery Expectations Survey (HFSS-KSE) in group AO-B

Item	*n*	Mean	Very	Somewhat	Little	Not
Pain relief	47	1.19	38 (81%)	9 (19%)	0	0
Improve ability to walk	45	1.19	39 (87%)	5 (11%)	0	1 (2%)
Confidence about the knee	50	1.24	22 (50%)	14 (32%)	5 (11%)	3 (7%)
Increase knee stability	47	1.28	36 (77%)	10 (21%)	0	1 (2%)
Improvement to be employed	47	1.28	37 (79%)	8 (17%)	1 (2%)	1 (2%)
Improve ability to climb stairs	45	1.29	34 (76%)	10 (22%)	0	1 (2%)
Stop knee from Catching/buckling	44	1.3	33 (75%)	10 (23%)	0	1 (2%)
Stop knee from stiffening	48	1.33	35 (73%)	11 (23%)	1 (2%)	1 (2%)
Back in intact status	50	1.36	35 (70%)	13 (26%)	1 (2%)	1 (2%)
To avoid future degeneration	48	1.38	33 (69%)	13 (27%)	1 (2%)	1 (2%)
Increase knee mobility	46	1.39	31 (67%)	13 (28%)	1 (2%)	1 (2%)
Improvement in activities in daily life	46	1.39	31 (67%)	13 (28%)	1 (2%)	1 (2%)
Improve ability to knee	45	1.49	27 (60%)	15 (33%)	2(4%)	1 (2%)
Improve ability to squat	44	1.5	27 (61%)	13 (30%)	3 (7%)	1 (2%)
Stop from giving way	43	1.51	25 (58%)	15 (35%)	2 (5%)	1 (2%)
Improve psychological well-being	44	1.66	27(61%)	10 (22%)	3 (7%)	4 (9%)
Ability to maintain health	44	1.68	25 (57%)	12 (27%)	3 (7%)	4 (9%)
Improve to interact socially	43	1.67	25 (58%)	10 (2%)	5 (12%)	3 (7%)
Improve ability to exercise	44	1.75	22 (50%)	14 (32%)	5 (11%)	3 (7%)
Improve ability to run	47	1.79	23 (49%)	15 (32%)	5 (11%)	4 (9%)

**Table 3:** Hospital for Special Surgery-Knee Surgery Expectations Survey (HFSS-KSE) in group AO-C

Item	*N*	Mean	Very	Somewhat	little	Not
Pain relief	61	1.18	50 (82%)	10 (16%)	1 (2%)	0
Improve ability to walk	59	1.19	53 (90%)	6 (10%)	0	0
Confidence about the knee	62	1.15	54 (87%)	7 (11%)	1 (2%)	0
Increase knee stability	57	1.26	43 (75%)	13 (22%)	1 (2%)	0
Improvement to be employed	58	1.36	42 (72%)	12 (21%)	3 (5%)	1 (2%)
Improve ability to climb stairs	57	1.26	43 (75%)	13 (23%)	1 (2%)	0
Stop knee from Catching/buckling	57	1.35	40 (70%)	15 (26%)	1 (2%)	1 (2%)
Stop knee from stiffening	60	1.18	50 (83%)	9 (15%)	1 (2%)	0
Back in intact status	63	1.59	37 (59%)	21 (33%)	4 (6%)	1 (2%)
To avoid future degeneration	61	1.43	37 (61%)	23 (38%)	1 (2%)	0
Increase knee mobility	57	1.30	42 (74%)	14 (25%)	1 (2%)	0
Improvement in activities in daily life	56	1.32	40 (71%)	14 (25%)	2 (4%)	0
Improve ability to knee	57	1.72	26 (46%)	22 (39%)	8 (14%)	1 (2%)
Improve ability to squat	58	1.53	32 (55%)	22 (38%)	3 (5%)	1 (2%)
Stop from giving way	59	1.42	37 (63%)	19 (32%)	3 (5%)	0
Improve psychological well-being	56	1.61	27 (48%)	25 (45%)	3 (5%)	1 (2%)
Ability to maintain health	54	1.63	27 (50%)	22 (41%)	3 (6%)	2 (4%)
Improve to interact socially	55	1.58	29 (53%)	21 (38%)	4 (7%)	1 (2%)
Improve ability to exercise	55	1.67	26 (47%)	21 (38%)	8 (15%)	0
Improve ability to run	57	1.79	26 (46%)	18 (32%)	12 (21%)	1 (2%)

### Residual pain

33 patients (65%) in group B and 29 patients (47%) in group C expected at most occasional pain during demanding sports (n.s.). In the collective, previous knee surgery showed no significant association with residual pain expectation between the two groups. For details see Table 4.

**Table 4:** Expectation concerning residual pain following osteosynthesis for proximal tibial fractures

Residual pain (*n*)	Fracture type AO-B	Fracture type AO-C
No pain at all	21 (41%)	18 (29%)
Occasional pain in demanding sport	11 (22%)	11 (18%)
Occasional pain in less demanding sport	13 (26%)	24 (38%)
Occasional pain in labour/daily activities	6 (12%)	10 (16%)

### Return to work and duration of inability to work postoperatively

**Fig. 1:**
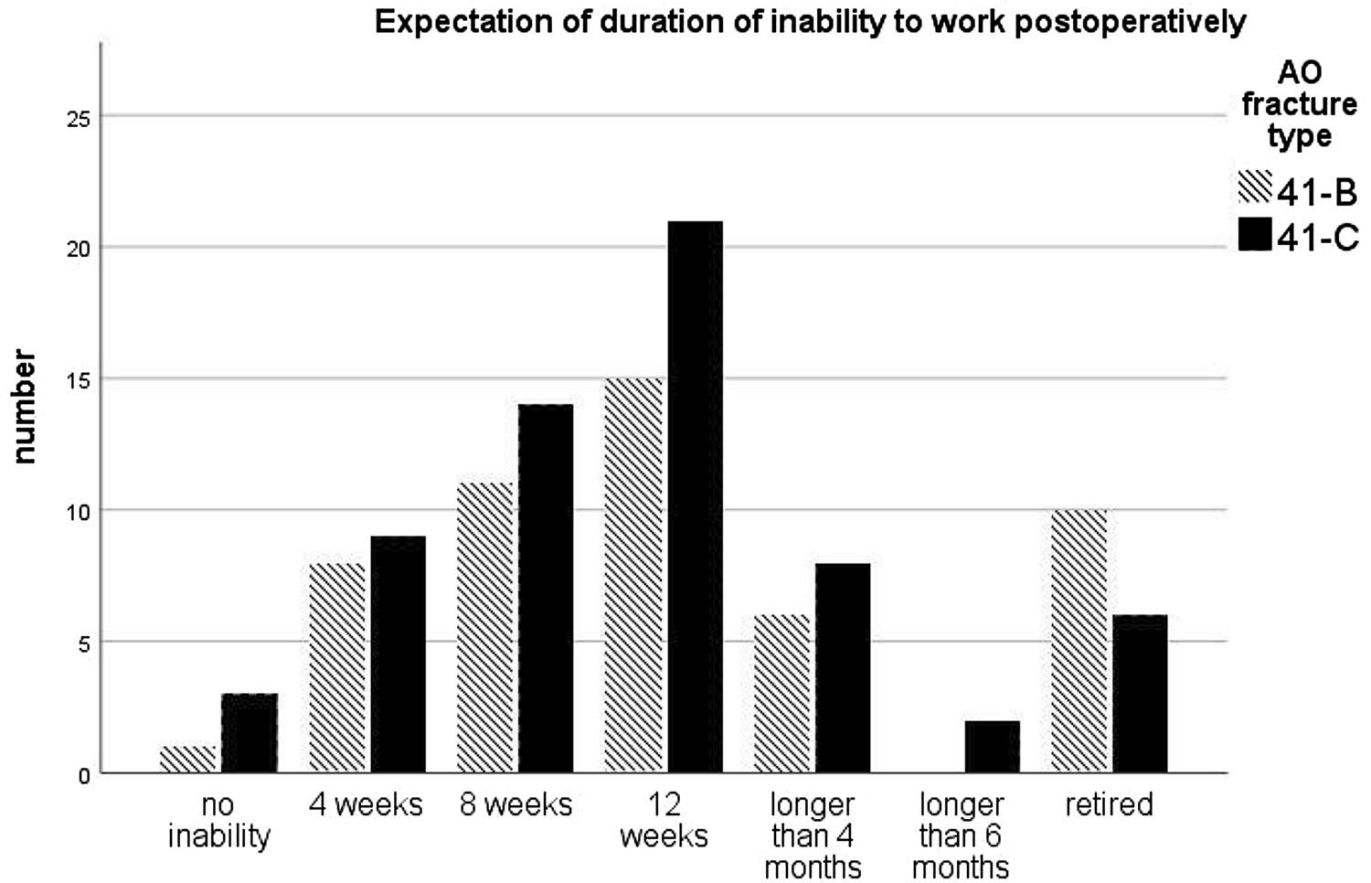
Expectation of duration of incapacity to work (weeks) after osteosynthesis in complex proximal tibial fracture

41 patients (80%) in group B and 57 patients (90%) in group C were employed. 16 patients (14%) were retired. All employed patients are expected to return-to-work. 34 patients (83%) in group B and 38 patients (67%) in group C expected return-to-work without limitations. Previous knee surgery did not affect the expectation (n.s.). Expected mean time to return-to-work was 9.7 weeks in group B and 10.1 weeks in group C (n.s.). 35 patients (85%) in group B and 47 patients (82%) in group C expected inability to work for at most 12 weeks. For details see Fig. 1.

### Return to sports

25 patients (49%) in group B and 21 (33%) patients in group C expected to return-to-sports without limitations. 11 patients (22%) of group B and 17 patients (22%) of group C expected return-to-sports with small limitations. There was no difference between groups (n.s.). Previous knee surgery correlated with lower return-to-sports expectations (*p* = 0.005).

### Comparing knee joint to healthy knee joint, risk for osteoarthritis, prevention/delay of knee replacement

**Fig. 2:**
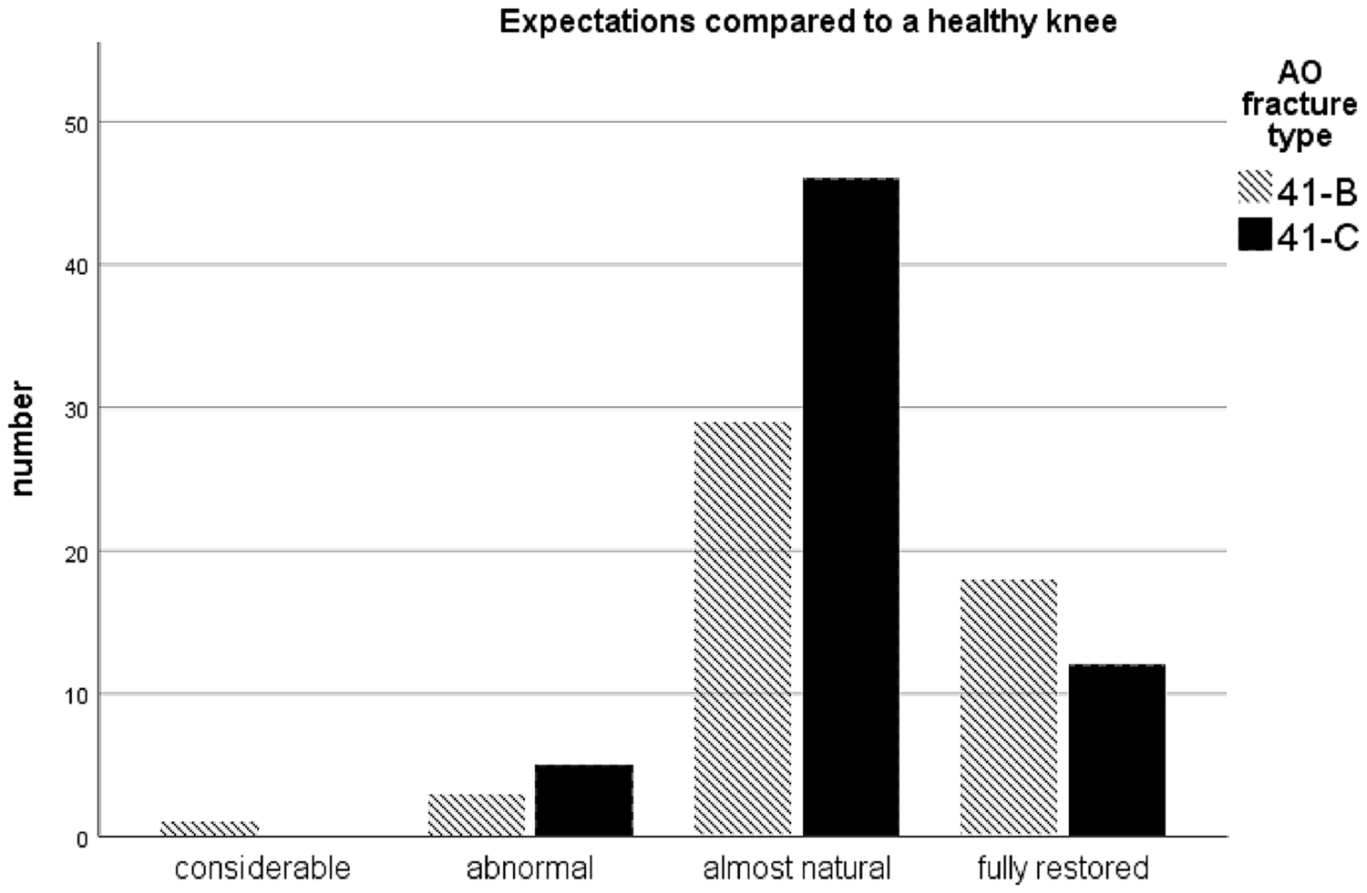
Expectations of comparing the injured knee to a healthy knee

92% (B: *n* = 47; C: *n* = 58) expected at least an almost natural joint and/or no differences to a healthy knee. For details see Fig. 2.

Regarding risk for secondary OA, 17 patients (33%) in group B and 14 patients (22%) in group C expect that surgery prevents an accelerated risk for developing OA. Only 3 (6%) patients of group B and 10 (16%) patients of group C expected, that surgery cannot prevent OA. There was no difference between groups (n.s.). For details see Table 5.

**Table 5:** Estimated risk for development of secondary osteoarthritis after osteosynthesis for complex proximal tibial fracture

Risk for osteoarthritis (*n*)	Fracture type AO-B	Fracture type AO-C
Surgery will prevent accelerated risk of osteoarthritis	17(33%)	14 (22%)
I expect an accelerated risk for osteoarthritis	22 (42%)	27 (43%)
I expect a considerably accelerated risk for OA	9 (18%)	12 (19%)
Surgery cannot prevent an accelerated risk for osteoarthritis	3 (6%)	10 (16%)

30 patients (59%) in group B and 33 patients (52%) in group C expected prevention of TKA by surgery. Only 7 (14%) patients in group B and 12 patients (19%) in group C did not expect preventive surgical effects. For details see Table 6.

**Table 6:** Expectation on prevention of total knee arthroplasty (TKA) by osteosynthesis for complex proximal tibial fracture

Prevention of knee replacement (*n*)	Fracture type AO-B	Fracture type AO-C
Full prevention	30 (59%)	33 (52%)
Delay of 15–20 years	8 (16%)	12 (19%)
Delay of 5–10 years	5 (10%)	5 (8%)
Delay of 1–4 years	1 (2%)	1 (2%)
No prevention	7 (14%)	12 (19%)

### Patients’ expectations depending on gender, age and workload

No difference could be shown between male/female patients regarding residual pain in both groups (n.s.). In groups B and C, no difference concerning return-to-work was shown (n.s.). Male patients in group B had significantly lower expectations to return-to-sports than women (*p* = 0.022). Concerning risk for secondary OA no difference was shown for gender in both groups (n.s.). In both groups, patients ≥ 50 years had lower expectations concerning residual pain (AO-B p = 0.04; AO-C *p* = 0.03). In group C mean expected time to return-to-work was 10.1 weeks for patients younger and older than 50 years (n.s.). In contrast, in group B younger patients expected a significantly shorter time to return-to-work than older patients (*p* = 0.05; 95% CI = − 0.006 to =5.21). In both groups younger patients had higher expectations to return-to-sports (AO-B p = 0.05; AO-C *p* = 0.009). The estimated risk for secondary OA was similar in both groups regardless of age (n.s.).

In patients with more demanding physical work, a negative correlation was shown for ability to knee (*r* = − 0.3), stop the knee from catching/buckling (*r* = − 0.3), improvement to be employed (*r* = − 0.3), and to avoid future degeneration (*r* = − 0.3). In both groups no difference could be shown for “residual pain” concerning low or high workload (n.s.). Patients with low work intensity expected a shorter time to return-to-work in both groups. In both groups, no correlation between return-to-sports and work, and/or risk for OA was shown (n.s.).

## Discussion

This study showed that patients overestimated the prognosis of osteosynthesis for complex proximal tibial fracture regardless of fracture type.

### Expectations concerning knee functions (HFSS-KSE Survey) and residual pain

The patients in this study had high expectations regarding restoring knee function regardless of fracture type. Both groups showed similar results concerning priorities of restoring knee function. All items on the four-point Likert Scale ranged between 1.18 and 1.79. “Pain relief” and “improve ability to walk” were rated to be of the highest priority in both groups.

Residual pain and limited knee function may appear after proximal tibial fractures [2]. They may influence and attenuate quality of life to a significant amount. Restricted knee function may lead to restricted sports ability or reduced workload. Residual pain influences not only the physical but also the emotional health, and thus may lead to reduced quality of life [10]. In this study, most patients expected residual pain at most occasionally during demanding sports. In a study by *Evangelopoulos *et al*.* evaluating mid-term results after proximal tibial fracture, 77% of the patients reported pain or discomfort in the affected knee joint within 56 months (mean) after proximal tibial fracture [11]. Severe injury of the tibia frequently leads to residual pain or limited knee function [12]. In this study, *Wirbel *et al*.* evaluated life quality after proximal tibial fracture. They found out that after proximal tibial fracture mean NRS (numeric rating scale) was 4.5, IKDC (International Knee Documentation Committee Subjective Knee Form) score was 50.46, and the EQ 5D (European Quality of Life 5 Dimensions) was 7.47.In the presented study more than 47% of the patients in both groups expected at most occasional pain when participating in demanding sports. Comparing the results of the study to the existing literature, expectation for residual pain was overestimated [11, 13, 14]. Additionally, regarding the HFSS-KSE items, restoring knee function and full range of motion had highest patient priority. The ability to walk determines decisively the independence of the human being. It is therefore understandable and obvious that, in addition to freedom from pain, the complete loading of the fractured leg is in the focus of patients´ expectations. Nevertheless, patients must be aware of functional limitations that can appear in mid- and long-term [15, 16]. Postoperatively, the range of motion may be significantly limited, and the functional outcome may be disappointing. This must be discussed with the patient unmistakably.

### Patients with higher workload expect longer incapacity to work

In both groups, most patients expected to return-to-work at most 12 weeks postoperatively without limitations. Only a small number expected a longer time, respectively limitations. It seems that patients with high demanding physical workload assessed the severity of their injury better, as they expected significantly longer incapacity to work. Similar to this study, *Kraus *et al*.* reported a mean time of 16 weeks to return-to-work. As well, longer incapacity to work was associated with more demanding physical workload [17]. Additionally, the study showed a post-injury shift to less demanding jobs and the reduction of working hours. Similar results were found in elective knee surgery e.g. for High Tibial Osteotomy (HTO) [18]. The data of the presented study suggests, that patients with high physical workload understand, that it will take more time to recover. Nevertheless, patients in this study underestimated their prognosis to return-to-work. Potential impairment in work intensity or working hours needs to be clearly discussed with the patient.

### Patients overestimate their prognosis regarding return-to-sports

Many patients of this study were young and active. In both groups most patients expected to return-to-sports with at most small limitations. In contrast to return-to-work, patients with previous knee surgery showed significantly lower expectations regarding return-to-sports. In comparison to the expectations formulated in this study, other studies showed, that return-to-sports was possible, but on a less demanding level [19, 20]. Loibl et al*.* showed that only around 50% of skiers returned to skiing after proximal tibial fracture [21]. In this study, fracture type showed a significant negative impact on the sportive outcome. In the presented study patients significantly overestimated their prognosis regarding return-to-sports.

### Patients underestimate their risk for secondary osteoarthritis and total knee arthroplasty

In a study by Honkonen et al*.* secondary OA after proximal tibial fracture was found in 36% in non-operated patients and in 50% of operated patients. In this study, 7 years following proximal tibial fracture, OA was found in 44% of all included cases [22]. Mid-term results following proximal tibial fracture (AO-C) showed at least mild signs of OA in more than half of the patients [23]. In the presented study, about 50% of the patients in both groups expected an accelerated or considerably accelerated risk for developing OA. 33% in group B and 22% in group C thought that surgery will prevent the risk to develop OA. In addition to these findings, more than 80% in both groups thought that surgery will at least delay need for TKA. Wasserstein et al. showed that 7% of patients with proximal tibial fracture had TKA after 10 years. This means a 5 times increased likelihood compared to a matched group of the general population [24]. In this study, older patients, and those with more severe fractures, had a higher risk for TKA. Increasing rates for the risk for TKA were found for patients older than 48 years. In the presented study with a mean age of patients of 48 years in AO-B, and respectively 50 years in AO-C, the risk to develop secondary OA and the need for TKA seems, therefore, to be underestimated.

In contrast to the studies already discussed, and their findings regarding reduced function and functionality, persistent pain, and long-term outcome, other studies show better outcomes after proximal tibial fracture. A study by Rohra et al. evaluating Schatzker V and VI fractures demonstrated good to excellent outcomes using The Knee Society Score and radiologic criteria [25]. However, this study describes a very small patient population (*n* = 34), and a short follow-up (minimum 3 years). A long-term study by Rademakers et al. of patients treated between 1975 and 1995, also shows very good functional and radiological results [26]. Although the initial number of patients of the study was very large (202 patients), long-term follow-up was only completed for half of the patients (119 patients). Secondary OA occurred in 11% of these patients.

The results of this study showed that trauma patients overestimate their prognosis after complex tibial fracture to a high amount regarding many fields of knee function, residual pain, return to work/sports, and the risk for secondary OA. It has already been shown in other orthopaedic specialities that patients have unrealistic expectations regarding surgery [5, 6]. This may influence satisfaction with the surgical outcome [27, 28]. Bearing this in mind, the results of this study should motivate surgeons to better instruct patients towards an enhanced comprehension of the acute medical condition and procedure. It is of utmost importance to direct patient education towards shared decision-making, and to provide a framework for setting reasonable goals. It must be considered the surgeons´ responsibility to understand this and guide patients towards sensible expectations. This is especially true in orthopaedic trauma surgery, with acute impairment and pain, combined with short notice decision-making and surgical treatment. We need to shift our patients away from impractical beliefs such as *no* than rather *less* pain, and *normal* than rather *improved* function. Meaning a joint may be “repaired”, but not necessarily be *normal,* following osteosynthesis for complex intraarticular fractures. Better instruction and information can be the key to improve patient comprehension, what must be considered a determinant of outcome.

### Limitations

This study has some limitations. Only patients undergoing surgery were included. Conservative treatment was not taken into account, as conservative treatment must be considered as no valuable option for this type of fractures. Written informed surgical consent was not standardised. This may influence patients´ expectations significantly. The surgical technique was not standardized. The questionnaires used for this study only evaluated subjective patients´ expectations without measuring objective outcome. For the subject of this study objective outcome measures were not the scope. To evaluate further on the subject, a follow-up study investigating on fulfillment of patient expectations would be necessary.

## Conclusion

Expectations on surgery for complex proximal tibial fracture are high regardless of fracture type. The prognosis of many health and lifestyle domains was overestimated. Risk for osteoarthritis was underestimated as well. This study should sensitize surgeons to discuss realistic expectations. This may help to improve patient comprehension, what leads to sensible expectations, resulting in improved patients´ satisfaction.
